# Response to commentary on “Examples of overlooking common sense solutions: the domestication gene and selection against mortality”

**DOI:** 10.3389/fgene.2014.00343

**Published:** 2014-09-30

**Authors:** P. Bijma, W. M. Muir, E. D. Ellen

**Affiliations:** ^1^Animal Breeding and Genomics Centre, Wageningen UniversityWageningen, Netherlands; ^2^Department of Animal Sciences, Purdue UniversityWest Lafayette, IN, USA

**Keywords:** indirect genetic effects, animal welfare, laying hens, cannibalism, quantitative genetics, breeding

In a commentary in Frontiers, Van Rooijen (2014) states: “Today the developments in genetics are exciting. Perhaps this explains why geneticists sometimes seem to overlook common sense solutions. One example of this is the selection experiment done by Bijma et al. (2007a,b). ……As a result their selection seemed not very efficient.” In those two papers, however, we do not report a selection experiment. The first paper presents general quantitative genetic theory, showing how interactions among individuals alter heritable variation in traits, and how this can affect response to selection. The second paper presents general methodology to estimate the quantitative genetic parameters for such traits, and illustrates this methodology using a population of laying hens showing high mortality due to pecking behavior. Neither of those papers report results of a selection experiment.

For the specific case of feather pecking, Van Rooijen suggests that the methodology would rest on the assumption that feather pecking results from aggression. This is not true. The strength of the methodology is that it captures the full heritable variance in the trait, irrespective of the underlying mechanism. Hence, for mortality due to pecking, the method captures both the actor component originating from the individual performing the pecking behavior and the victim component, as well as their covariance. These components are identified statistically from the covariances between trait values of relatives and their social partners, without any assumption on the underlying mechanisms. The method produces optimal breeding values, given the genetic parameters. Results of selection experiments based on the theory, presented in other papers (Muir, 1996; Muir et al., 2013; Ellen et al., 2014), confirm the efficiency of the proposed methodology.

## Conflict of interest statement

The authors declare that the research was conducted in the absence of any commercial or financial relationships that could be construed as a potential conflict of interest.
